# Inhibitory networks of the amygdala for emotional memory

**DOI:** 10.3389/fncir.2013.00129

**Published:** 2013-08-01

**Authors:** Seungho Lee, Su-Jeong Kim, Oh-Bin Kwon, Joo Han Lee, Joung-Hun Kim

**Affiliations:** Department of Life Science, Pohang University of Science and TechnologyPohang, South Korea

**Keywords:** neural circuits, inhibitory neurons, amygdala, fear, extinction

## Abstract

The amygdala is important for emotional memory, including learned fear. A number of studies for amygdala neural circuits that underlie fear conditioning have elucidated specific cellular and molecular mechanisms of emotional memory. Recent technical advances such as optogenetic approaches have not only confirmed the importance of excitatory circuits in fear conditioning, but have also shed new light for a direct role of inhibitory circuits in both the acquisition and extinction of fear memory in addition to their role in fine tuning of excitatory neural circuitry. As a result, the circuits in amygdala could be drawn more elaborately, and it led us to understand how fear or extinction memories are formed in the detailed circuit level, and various neuromodulators affect these circuit activities, inducing subtle behavioral changes.

## INTRODUCTION

The amygdala is an almond-like structure that is located within the temporal lobe of the brain, adjacent to the ventral hippocampus. The neural circuits of the amygdala and its connected brain areas are thought to be critically important for emotional learning, especially the formation and storage of fear memories (LeDoux, 2003). Formation and recall of these emotional memories are essential for animal survival. Therefore, the behavioral features and signaling mechanisms of fear memories are highly conserved across the animal realm. The neuronal engrams that permit storage of learned fear are formed almost immediately upon exposure of the subject to threats, and are represented by apparent changes in behavioral patterns. Thus, this perspicuity of fear responses provides opportunities to investigate both the physiological functions of amygdala circuits and the molecular and cellular mechanisms that underlie long-lasting memories.

**FIGURE 1:**
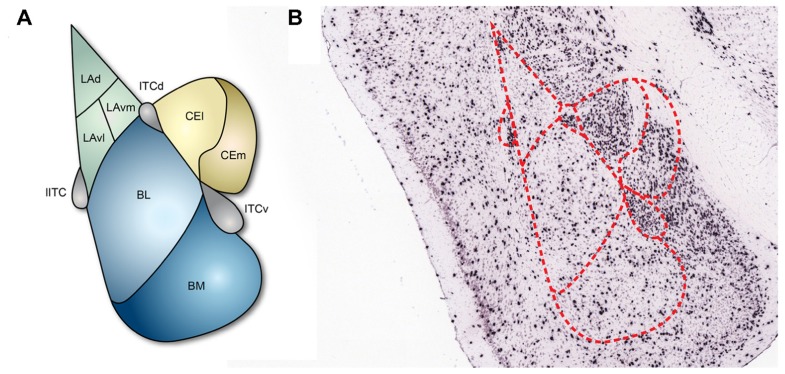
**Subnuclei of the amygdala and inhibitory neurons**.(A) The basic structure of the amygdala. Cortex-like subnuclei (blue) are located laterally, while striatum-like subnuclei (yellow) are located medially. ITCs (purple) reside at the edge of BLA. LA, lateral amygdala; BL, basolateral nucleus; BM, basomedial nucleus; CEl, lateral part of central amygdala; CEm, medial part of central amygdala; lITC, lateral paracapsular intercalated cell mass; ITCd, dorsal part of intercalated cell mass; ITCv, ventral part of intercalated cell mass. **(B)** *In situ* hybridization showing the 67-kDa isoform of the GABA synthesizing enzyme glutamic acid decarboxylase (GAD67) in coronal brain slices (Allen Institute for Brain Science, 2012). The GAD67 expression profile indicates that inhibitory neurons are sparsely present in the cortex-like subnuclei and densely present in ITCs and the striatum-like subnuclei.

The amygdala is composed of several subnuclei that can be largely classified into two groups, cortex- and striatum-like structures (Ehrlich et al., 2009; **Figure 1**). The basolateral amygdala (BLA), which includes the lateral amygdala (LA) and the basal amygdala (BA), would be considered to be cortex-like nuclei since the structural organization and cellular composition of these structures are similar to those of the cortex: the majority of cells are excitatory projection neurons and only a minority of cells are interneurons that form local inhibitory circuits (Carlsen, 1988; Smith and Pare, 1994). BA can be further divided into the basolateral nucleus (BL) and the basomedial nucleus (BM; **Figure 1**). By contrast, the central nucleus of the amygdala (CEA) is a striatum-like nucleus. CEA is located medial to BLA and mostly consists of GABAergic neurons (McDonald, 1982) with morphological, physiological, and biochemical properties that resemble medium spiny neurons in the striatum (Martina et al., 1999; Schiess et al., 1999; Markram et al., 2004; Ascoli et al., 2008). CEA is further divided into lateral (CEl) and medial (CEm) parts, which serve different functions and have distinct connectivity (Ciocchi et al., 2010). In addition, the amygdala contains multiple intercalated cell masses (ITCs), which are thought to regulate the interconnectivity between the distinct amygdala subnuclei and also between the amygdala and extra-amygdala structures. ITCs are composed of GABAergic neurons (Busti et al., 2011) and are located along the borders of the BLA. Depending on the residing loci, ITCs are named as the lateral paracapsular (lITC), the dorsal (ITCd), and the ventral (ITCv) ITC (**Figure 1**). Several studies have demonstrated that the individual ITCs provide feedforward inhibition and control expression of fear responses (Royer et al., 1999; Marowsky et al., 2005).

Despite these well-defined amygdala circuits and connections, it has not been well-understood yet how fear memories are stored, processed, and extinguished particularly at the circuit level. This lack of understanding was mainly due to the absence of methodologies that allow temporal and spatial manipulations of select circuit components. However, the recent advent of optogenetics provides valuable tools for researchers to address these circuit level questions with unprecedented temporal and spatial specificities. By combining optogenetic tools with other experimental paradigms, such as *in vitro* and *in vivo* recordings, recent studies have confirmed and further substantiated that excitatory amygdala connections and dynamic changes in the synaptic strength can modulate various phases of fear memories. Furthermore, a number of studies provide evidence that inhibitory elements of amygdala circuits play major roles in defining and processing of fear engrams and fear memories. Herein, we provide an overview of these new findings that suggest the functional importance of amygdala inhibitory circuits in acquisition, expression, and extinction of fear memories.

## NEURAL CIRCUITS THAT UNDERLIE FEAR MEMORIES

Fear conditioning is a behavioral paradigm that has been widely used to probe associative fear learning. During fear conditioning, an unconditioned stimulus (US), such as an electrical foot shock, is simultaneously presented with a neutral conditioned stimulus (CS), such as a tone or a light, to subjects. The subjects learn to predict aversive events and exhibit fear responses not just to the US, but also to the CS. Mechanically, sensory information encoding the CS and the US converges in LA, which results in long-term potentiation (LTP) at LA excitatory synapses (Rogan et al., 1997). This LTP enables the CS alone to elicit fear responses (freezing behavior) normally observed only when subjects confront threats (LeDoux, 2000). However, when the CS is presented repeatedly without the US, the ability to elicit fear responses is diminished. This phenomenon is called fear extinction (Maren and Quirk, 2004). Numerous studies have demonstrated that fear extinction does not actually erase the existing fear memories, but instead results from formation of *de novo* memories that suppress the behavioral expression of the previously learned fear (Rescorla, 2001; Bouton et al., 2006).****

Sensory information pertinent to the CS and the US is initially integrated and processed in LA. Thus, LA functions as the synaptic interface of the amygdala, receiving sensory signals from various sources such as thalamic and cortical inputs. Thalamic inputs usually deliver rapid but unprocessed information, whereas cortical inputs convey relatively delayed but processed information from visual, auditory, or somatosensory cortices (Li et al., 1996b; LeDoux, 2000). Although thalamic and cortical inputs are thought to convey different aspects of sensory information (unprocessed or processed, respectively), they reach same components in LA (Szinyei et al., 2000). That is, pyramidal neurons and local inhibitory neurons in LA receive signals from thalamic inputs as well as cortical inputs (Li et al., 1996b; Szinyei et al., 2000). Both thalamic and cortical inputs form feedforward inhibition onto pyramidal neurons through local inhibitory neurons (Ehrlich et al., 2009). Interestingly, cortical inputs are innervated to lITCs residing in the external capsule and in turn, lITC also provides feedforward inhibition onto LA pyramidal neurons (Marowsky et al., 2005). Thus, both lITC neurons and LA local inhibitory neurons participate in suppression of LTP in the temporal association cortex (TeA)–LA pathway (Morozov et al., 2011). Importantly, activity of local inhibitory neurons alone without that of lITC neurons was not sufficient to produce complete inhibition of LTP in the TeA–LA pathway (Morozov et al., 2011), suggesting the importance of lITC in modulating cortica inputs.

Following association and processing of CS and US signals in LA, the newly integrated information is transferred to BA, and then CEA. Finally, the neural activity of CEm determines fear responses. This LA–BA–CEA pathway is a major route for flow of sensory signals, leading to fear memories. A subset of BA neurons is then recruited based on the physiological characteristics of the transferred signals (Herry et al., 2008; Amano et al., 2011). Approximately 14% of BA neurons respond to fear-inducing cues, which can be referred to as fear neurons (Herry et al., 2008; Amano et al., 2011), although the exact contribution of these BA neurons to the fear behavior remains to be clarified. A subregion of medial prefrontal cortex (mPFC), the prelimbic cortex (PL), is also critical for expression of fear-related behavior (Corcoran and Quirk, 2007). CS-evoked unit activity in PL increases both during and following fear conditioning, but subsequently decreases after fear extinction (Burgos-Robles et al., 2009). Activity of PL neurons during CS presentation is thought to be originated from activity of BLA because the CS-evoked activity in PL is regulated by BLA (Sotres-Bayon et al., 2012). Notably, PL has reciprocal connections with the amygdala, especially with BA (Vertes, 2004; Likhtik et al., 2005; Herry et al., 2008). Hence, it is conceivable that PL would control fear expression by modulating activity of BA. Although it should be further clarified which pathways are responsible for serial activation of LA–BA–PL–BA–CEm, BA would stimulate CEm through interaction with PL, which results in fear responses (Pitkanen et al., 1997; Vertes, 2004; Likhtik et al., 2005).

Although sensory information related to fear appears to be primarily relayed via the LA–BA–CEA pathway described above, other pathways have been inferred based on BA pre-training lesion studies. In these experiments, fear acquisition is unaffected despite BA lesions prior to fear conditioning (Amorapanth et al., 2000; Goosens and Maren, 2001; Nader et al., 2001). Therefore, other synaptic connections, such as the LA–CEl–CEm pathway that involves extensive inhibitory connectivity, seem to also contribute to fear memories (Ciocchi et al., 2010; Haubensak et al., 2010; Tye et al., 2011).

## MODULATORY ROLES OF INHIBITORY CIRCUITS IN ACQUISITION AND EXPRESSION OF FEAR MEMORIES

The vast majority of published studies have focused on synaptic changes and plasticity of excitatory connections in the amygdala during fear conditioning in order to elucidate the neuronal substrates of emotional learning and memory. However, emerging evidence indicates that inhibitory circuits containing interneurons are critical components of neural networks that control neuronal activity and animal behavior. In the amygdala, inhibitory circuits play major roles in controlling the expression and extinction of fear memories (Herry et al., 2010; Tye et al., 2011). The inhibitory elements in the amygdala circuits were initially thought to control neural activity simply by inhibiting excitatory transmission. However, the precise roles of the inhibitory circuits are much more complex and instructive than previously thought. As the major input station for the amygdala, LA receives synaptic inputs from a number of brain regions. Connections to LA projection neurons are basally suppressed by inhibitory circuits in the amygdala in normal conditions (Lang and Pare, 1998; Bissiere et al., 2003; Woodruff and Sah, 2007). However, if the organism is exposed to fearful situations, then inhibition is released and the resulting decrease in synaptic inhibition of LA projection neurons permits occurrence of LTP and associative learning (Bissiere et al., 2003). Neither LTP nor acquisition of fear memories occurs without disinhibition (Li et al., 1996a; Lang and Pare, 1997; Bissiere et al., 2003). Inhibitory circuits in BLA are also involved in the shaping of fear memories. There are a number of synaptic inputs impinging onto LA projection neurons, and only the salient signals that are above a specific intensity should induce LTP and fear memories. The trivial noise signals should be disregarded with high fidelity. Tonic suppression derived from local inhibitory neurons helps the circuit to ignore these unimportant signals. Thus, the tonic effects of inhibitory neurons are essential for specific stimulation of LA projection neurons and encoding of precise memories. Several studies have supported active roles of inhibitory circuits by showing that specific fear memories are not formed in the absence of inhibitory components. For instance, deletion of presynaptic GABA_B_ receptors from glutamatergic inputs projecting onto LA projection neurons facilitates non-associative homosynaptic LTP in the cortical pathway and results in fear generalization that is an important characteristics of anxiety disorders (Shaban et al., 2006). Likewise, animals that are deficient in the 65-kDa isozyme of glutamic acid decarboxylase (GAD65) or the GABA_A_ receptor α1 subunit also exhibit fear generalization behavior (Bergado-Acosta et al., 2008; Wiltgen et al., 2009). Thus, basal inhibitory circuits serve to increase the signal-to-noise ratio for salient inputs during fear conditioning and ultimately to enhance the specificity of neural activity toward the salient events.

## INHIBITORY CIRCUITS RECRUITED FOR FEAR AND EXTINCTION MEMORIES

Inhibitory circuits are not only involved in tuning excitatory transmission, but are also active components of intra-amygdala fear pathways necessary for acquisition and extinction of fear memories. More specifically, inhibitory circuits control fear behavior during acquisition and expression of fear memories and construct new suppressive memories during fear extinction.****

### INHIBITORY CIRCUITS INVOLVED IN ACQUISITION AND EXPRESSION OF CONDITIONED FEAR

**FIGURE 2:**
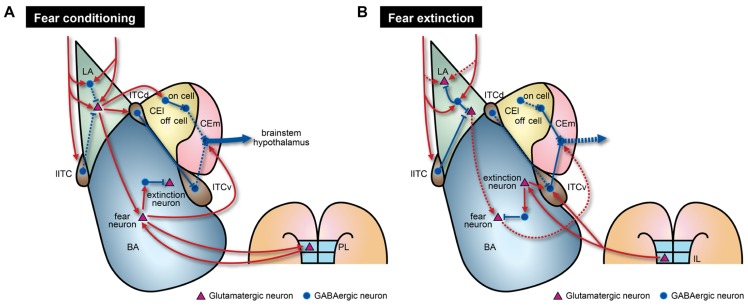
**Model of amygdala circuitry during fear conditioning and extinction.** The model contains both known and hypothetical neural connections. Solid and dashed lines indicate strengthened and weakened connections, respectively, during the learning processes. **(A)** During fear conditioning, CEm output activity is enhanced by two intra-amygdala pathways. First, there are enhanced excitatory signals that originate from LA. In LA, there is disinhibition of local inhibitory circuits due to the effects of several biogenic amines. This disinhibition leads to increased neural activity in BA, ITCd, and CEl. Fear neurons in BA may receive direct excitatory inputs from LA. Augmented activity of fear neurons, mediated through reciprocal connections with PL regions, is necessary to activate CEm output neurons. Second, strong inhibition of CEloff neurons and signaling from ITCv to CEm output neurons are reduced by the LA–CElon–CEloff–CEm and LA–ITCd–ITCv–CEm pathways, respectively. **(B)** During fear extinction, enhanced neural activity in LA is reduced by increased suppression from local inhibitory circuits and depotentiation of glutamatergic synapses. Decreased connectivity between extinction-resistant neurons in LA and fear neurons in BA could also contribute to fear extinction. Furthermore, IL cortex sends glutamatergic projections to ITCv and possibly extinction neurons of BA. These IL projections may participate in suppression of BA fear neurons via local inhibitory circuits.

Besides adjusting strength of excitatory connections, inhibitory circuits are actively involved in acquisition of fear memories. Emerging evidence indicates that the inhibitory components of the amygdala play causal roles in the acquisition of fear memories (Goosens and Maren, 2003; Wilensky et al., 2006; Zimmerman et al., 2007). Inactivation of CEA impairs acquisition of fear memories, and overtraining can restore fear memories in the absence of BLA activity (Wilensky et al., 2006; Zimmerman et al., 2007; Ciocchi et al., 2010). CEA receives direct projections from the auditory cortex and the thalamus (Ledoux et al., 1987; Turner and Herkenham, 1991; McDonald, 1998) and NMDAR-dependent LTP can be induced in CEA (Samson and Pare, 2005). Recent studies provide evidence that fear conditioning induces activity-dependent synaptic plasticity within CEl (Ciocchi et al., 2010; Haubensak et al., 2010; Duvarci et al., 2011). Interestingly, two distinct groups of CEl neurons exhibit opposite responses to CS presentations, especially after fear conditioning. CElon cells are activated and CEloff cells are inhibited when a CS is presented (Ciocchi et al., 2010; Duvarci et al., 2011; **Figure 2A**). Although the underlying molecular mechanisms and functional significance remain unknown, CElon and CEloff cells can also be distinguished by expression of a specific signaling molecule, protein kinase C-δ (PKC-δ). PKC-δ is expressed in CEloff cells but not in CElon cells (Haubensak et al., 2010). Furthermore, activated CElon cells appear to suppress CEloff cells that normally lead to tonic inhibition of CEm neurons (**Figure 2A**). Thus, increased activity of CElon cells in response to a CS results in excitation of CEm neurons through disinhibition (Haubensak et al., 2010). However, CEloff cells show only basal activity during fear extinction (Duvarci et al., 2011), despite the reciprocal inhibitory connections of CElon/off cells (Ciocchi et al., 2010), suggesting that CEloff cells do not actively participate in fear extinction. Therefore, the LA–CEl–CEm amygdala circuits responsible for disinhibition may contribute to acquisition of fear memories (**Figure 2A**). LA also sends excitatory signals to ITCd neurons, which in turn inhibit CEl neurons (Pare and Duvarci, 2012). Moreover, ITCd sends projections to ITCv, which inhibits CEm neurons (Pare and Smith, 1993; Royer et al., 2000; **Figure 2A**). Supporting the inhibitory functionality from ITCd to ITCv, fear conditioning enhances expression of activity markers in ITCd, but not in ITCv (Busti et al., 2011). Taken together, fear acquisition would involve several circuits that include multiple inhibitory components such as the LA–BA–CEm, LA–CEl–CEm, and LA–ITCd–ITCv–CEm pathways (**Figure 2A**). These various pathways may be necessary for providing modulatory interfaces that confer distinct features to fear responses depending on diverse circumstances.

### INHIBITORY MEMORIES DURING FEAR EXTINCTION

When learned fear is repeatedly retrieved without presentation of US, the fear responses to CS rapidly decrease. The reduction of fear responses is attributable to formation of new suppressive memories, a process known as fear extinction (Herry et al., 2010). After successful fear extinction, CS cues that previously induced fear responses will no longer cause alterations in neural activity at the circuit level. Intriguingly, extinction of fear memories displays several characteristics that differ from those of fear acquisition and expression. First, the extinguished fear memories tend to recover spontaneously over time (Rescorla, 2004). Second, renewal of fear memories can occur in new contexts (Bouton, 2004). Finally, fear responses to a CS can be reinstated simply by re-exposure to the US alone (Rescorla and Heth, 1975). These features provide substantial evidence that existing fear memories *per se *do not seem to be erased by fear extinction because removed memory cannot return, but instead, new memories that decouple the CS from the US are formed (Rescorla, 2001; Bouton et al., 2006). Therefore, fear extinction is likely to recruit inhibitory circuits to suppress expression of the existing fear memories.

Conditioned stimulus-evoked unit activity in LA neurons increases during fear conditioning and returns to basal levels after fear extinction in a context-dependent manner (Quirk et al., 1995; Hobin et al., 2003). Although we cannot fully exclude the possibility that this decreased LA neuronal activity may be due to depotentiation of thalamo-amygdala synapses by extinction training (Kim et al., 2007), it would result from actions of inhibitory circuits that are newly recruited by the context signals. Hence, the newly recruited inhibitory circuits could then suppress fear-related physiological changes and behavior (**Figure 2B**). Importantly, extinction training does not quantitatively fully normalize the neuronal activity to the levels observed prior to fear conditioning. In fact, some LA neurons exhibit elevated activity that is resistant to fear extinction (Repa et al., 2001; An et al., 2012), and cue-dependent fear responses are not extinguished if BA is rendered inactivated during the extinction period (Herry et al., 2008; Sierra-Mercado et al., 2011). Collectively, reductions in LA neuronal activity alone do not seem to be sufficient for expression of fear extinction memories. Decreases in LA neuronal activity may be consequence of extinction-induced activation of inhibitory circuits that project to and affect a select subset of LA neurons.

Recent studies revealed that BA neurons also participate in acquisition of fear extinction memories. A subset of BA neurons (~17%) exhibits selective increase in CS-evoked activity during fear extinction training (Herry et al., 2008; Amano et al., 2011). Thus, they are referred to as extinction neurons that differ from BA fear neurons that show increased activity during fear conditioning, as described above (Herry et al., 2008; Amano et al., 2011; **Figure 2**). The two classes of neurons display largely distinct spiking activity in response to a CS, and therefore may discriminate between extinguished and non-extinguished stimuli. In support of this notion, the extinction neurons have extra-amygdala connectivity that differs from the fear neurons. BA extinction neurons receive innervations from mPFC, possibly the infralimbic region (IL; Vertes, 2004; Herry et al., 2008; **Figure 2B**), a region that normally promotes fear extinction. Taken together, two populations of BA neurons seem to belong to different circuits, but this observation merits further investigation to elucidate exact intra-amygdala circuitry containing input or output connections of these two types of BA neurons.

Indeed, CS-evoked firing of IL neurons increases during fear extinction training, and selective stimulation of IL during training facilitate extinction of fear responses (Milad and Quirk, 2002; Vidal-Gonzalez et al., 2006). Importantly, inactivation of IL prior to extinction training impairs retention of extinction memories whereas it does not affect acquisition of fear memories within a session (Laurent and Westbrook, 2009; Sierra-Mercado et al., 2011). Therefore, burst firing of IL neurons during extinction learning and innervations to BA extinction neurons may contribute to retention and expression of extinction memories. As mentioned above, inactivation of either BA or IL during extinction training session impaired the formation of extinction memories, which represents the interplay between two regions necessary for fear extinction. Moreover, IL sends projections to multiple subnuclei of the amygdala including BLA, CEA, and ITC. More specifically, IL projections are targeted to ITCv but not ITCd. A recent study indicated that ITCv neurons directly inhibit the neural activity of CEm neurons, which is consistent with the staining experiment showing enhanced neural activity in ITCv neurons after extinction training (Busti et al., 2011). ITCv receives augmented excitatory inputs from BA after extinction training (Amano et al., 2010; **Figure 2B**). Notably, there are marked deficits in expression of extinction memories when ITCv is lesioned (Likhtik et al., 2008). These suggest that inhibitory neurons in ITCv are required for normal retention and expression of fear extinction memories, possibly through feedforward inhibition of CEA neurons. Collectively, it is fairly reasonable to speculate that fear extinction memories require augmented transmission from BA to ITCv and that IL modulates synaptic transmission by regulating both the ITCv and BA extinction neurons (**Figure 2B**). Although further studies are required to better understand extinction mechanisms, the key elements for fear extinction should include recruitment of ITCv circuits that directly inhibit CEm output neurons in addition to other previously proposed mechanisms such as depotentiation of LA synapses (Kim et al., 2007), transitions from fear neurons to extinction neurons in BA (Herry et al., 2008; Amano et al., 2011), dynamic activity of CElon neurons (Duvarci et al., 2011), and enhanced activity of IL after extinction training (Milad and Quirk, 2002).

## REGULATORY FACTOR-MEDIATED ALTERATIONS OF INHIBITORY CIRCUITS

Inhibitory connections and their strength are controlled in a temporal fashion by regulators such as biogenic amines and neuropeptides. Thus, the inhibitory components of amygdala circuits serve as an interface for modulation by these regulators, which would define the dynamic features of inhibitory circuits for processing sensory information.

### BIOGENIC AMINE-MEDIATED CONTROL OF INHIBITORY CIRCUITS

**FIGURE 3:**
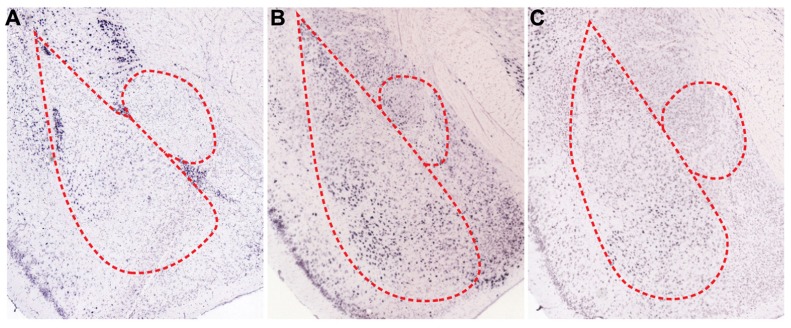
**Spatial expression patterns of regulatory receptors in the amygdala.** *In situ* hybridizations for various regulatory receptors expressed in the amygdala (Allen Institute for Brain Science, 2012). **(A)** Dopamine D_1_ receptors (D1Rs) are mainly expressed in ITCs and are sparsely expressed in BL. Both serotonin C_2_ receptors (5HT_2c_) **(B)**, and α-1D adrenergic receptors (α1D-AR) **(C)** are expressed in BLA, especially in LA and BM.

A number of biogenic amines regulate fear responses by affecting inhibitory elements. Dopamine (DA) is a well-known biogenic amine that controls inhibitory circuits and fear responses. In fact, dopaminergic axons that target inhibitory interneurons release DA when animals are subjected to stressful situations, such as electric foot shock (Inglis and Moghaddam, 1999; Yokoyama et al., 2005; Brischoux et al., 2009). At the circuit level, DA is intimately involved in gating synaptic plasticity and acquisition of fear memories (Rosenkranz and Grace, 2002; Bissiere et al., 2003). Released DA decreases inhibitory circuit-mediated suppression of BLA. In the thalamic pathway, DA reduces feedforward inhibition from local interneurons to LA projection neurons via activation of dopamine D_2_ receptors (D2Rs), which eventually allows for LTP induction at thalamo-amygdala synapses (Bissiere et al., 2003). LA projection neurons also receive potent feedforward inhibition by lITC when cortical inputs are stimulated (Marowsky et al., 2005). Mechanistically, activation of dopamine D_1_ receptors (D1Rs) significantly reduces the cellular excitability of lITC neurons through opening of G protein-coupled inwardly rectifying potassium channels (GIRKs) and subsequent inhibition of GABA release from axon terminals (**Figures 2A and 3A**). Thus, reduced feedforward inhibition from local LA interneurons and lITC GABAergic neurons enhances the activity of LA projection neurons and allows occurrence of LTP. Consistent with the gating effect of DA, blockade of DA receptors in the amygdala interferes with acquisition and expression of fear memories (Lamont and Kokkinidis, 1998; Guarraci et al., 1999, 2000; Greba and Kokkinidis, 2000; Greba et al., 2001). Therefore, synaptic plasticity in BLA is gated and adjusted by amygdala inhibitory circuits and DA is critically involved in shaping the firing patterns of BLA neurons by acting on the inhibitory components. On the other hands, DA is able to directly increase the excitability of BLA pyramidal neurons and fast-spiking BLA interneurons, which further amplify salient signals over the specific threshold (Kroner et al., 2005). Therefore, DA released during fear conditioning also enhances the signal-to-noise ratio, which helps amygdala circuits determine meaningful information. Supporting this hypothesis, animals show generalized anxiety phenotypes after foot shock conditioning when activation of dopaminergic neurons in response to aversive stimuli is genetically attenuated (Zweifel et al., 2011). Overall, inhibitory neurons mediate effects of DA on fear memories, including the gating of synaptic plasticity and shaping of cellular firing patterns. These effects contribute to the formation of more precise fear memories.

Serotonin (5-HT), another major biogenic amine, is intimately involved in controlling fear memories. BLA receives dense serotonergic projections from the dorsal raphe nucleus and also expresses multiple subtypes of 5-HT receptors (Sadikot and Parent, 1990; **Figure 3B**). A majority of published studies show that 5-HT mediates inhibitory effects on fear responses, especially during the expression phase (Hashimoto et al., 1996; Li et al., 2006; Montezinho et al., 2010). At the cellular level, iontophoresis of 5-HT into LA inhibits excitation of LA projection neurons (Stutzmann et al., 1998). Thus, the inhibitory role of 5-HT is likely mediated through actions on local GABAergic neurons and/or inhibitory connections from ITC, as previously suggested (Stutzmann and LeDoux, 1999).

Norepinephrine is also important for processing sensory information related with fear memories. LA receives tonic release of norepinephrine from the locus coeruleus, and noxious stimuli such as foot shock result in the phasic release of norepinephrine (Quirarte et al., 1998; Tanaka et al., 2000; Aston-Jones and Cohen, 2005). In fact, various subtypes of adrenergic receptors are present in BLA (**Figure 3C**), and infusion of norepinephrine into BLA promotes consolidation of fear memories (LaLumiere et al., 2003). However, inhibition of α1-subtypes adrenergic receptors in LA facilitates fear conditioning (Lazzaro et al., 2010), which suggests that each subtype of adrenergic receptors may play different roles for fear learning. The behavioral actions of norepinephrine may result from effects on inhibitory circuits, which allows for the occurrence of LTP. Indeed, norepinephrine reduces the excitability of local interneurons in LA and as a result, decreases GABAergic feedforward inhibition onto thalamic inputs (Tully et al., 2007). Taken together, these actions of norepinephrine permit LTP in the thalamic pathway (Tully et al., 2007).

### NEUROPEPTIDE-MEDIATED CONTROL OF INHIBITORY CIRCUITS

Neuropeptides are involved in regulation of inhibitory circuits, but the underlying mechanisms are not fully understood. For instance, vasopressin and oxytocin affect fear responses in an opposite manner (Carrasco and Van de Kar, 2003; Holmes et al., 2003; Amico et al., 2004; Gulpinar and Yegen, 2004; Griebel et al., 2005). Vasopressin administrated into CEA increases the neural activity (Lu et al., 1997; Huber et al., 2005), which results in increased expression of fear responses (Gulpinar and Yegen, 2004). However, enhanced expression of oxytocin decreases fear behavior (McCarthy et al., 1996). Despite the similarity on cellular effects (Raggenbass, 2001), their behavioral consequences were opposite. It could be explained from comprehension about circuits in CEA. That is, distinct population of CEl neurons expressing oxytocin receptors gives direct inhibition onto vasopressin receptor-expressing CEm neurons (Huber et al., 2005). As a result, activation of oxytocin receptors in CEl neurons inhibits the activity of CEm neurons (Viviani et al., 2011; Knobloch et al., 2012). Thus, vasopressin in CEm increases fear expression and oxytocin in CEl produces the opposite effect (Lu et al., 1997; Huber et al., 2005; Viviani et al., 2011; Knobloch et al., 2012). Furthermore, oxytocin receptor expressing neurons suppressed only periaqueductal gray (PAG) projecting CEm neurons which is related to freezing behavior, but not the dorsal vagal complex (DVC) projecting neurons related to increasing heart rate (Viviani et al., 2011). It provided us a good example how action of neuropeptide onto complex inhibitory circuits could modulate behavior precisely and produce diverse behavioral responses.

Stressful conditions can alter animal behavior through facilitated release of neuropeptides into the amygdala. When animals undergo withdrawal from chronic alcohol administration, the corticotrophin-releasing factor (CRF) system is activated. Injection of a CRF antagonist into CEA reverses anxiety-like behavior that is mediated by amygdala circuits (Rassnick et al., 1993). By contrast, neuropeptide Y (NPY) in the amygdala exerts potent anxiolytic effects (Heilig et al., 1993). NPY receptors are highly enriched in CEA and BLA (Tasan et al., 2010), and anxiety-like behavior following ethanol withdrawal is decreased when NPY is virally expressed in CEA (Primeaux et al., 2006). Furthermore, infusion of NPY into the amygdala decreases expression of learned fear and enhances fear extinction (Gutman et al., 2008). Interestingly, although they produce largely opposite behavioral outcomes, the CRF and NPY systems appear to converge on inhibitory neurons in CEA (Gilpin, 2012). Thus, it is fairly possible that CRF and NPY cause distinct behavioral effects by regulating the inhibitory neurons in the amygdala in opposite directions. Indeed, neuropeptide S (NPS) regulates inhibitory circuits and fear responses. When glutamatergic inputs to ITC are increased through activation of presynaptic NPS receptors, fear expression is reduced and fear extinction is promoted (Jungling et al., 2008). Collectively, these observations suggest that neuropeptides intimately control amygdala-mediated behavior by acting on inhibitory components of CEA.

Learned fear is modified by inhibitory circuits when the internal or external circumstances are changed. Various neuropeptides are released into CEA when animals are subjected to stressful conditions, and the inhibitory neurons express the receptors for these signaling molecules (Cassell et al., 1999; Gilpin and Roberto, 2012). Indeed, neuropeptides are involved in subtle control of fear responses, especially expression of fear memories (Bowers et al., 2012). Fear behavior can be immediately elicited by the neural activity of CEm, which is controlled by CEl inhibitory neurons (Tye et al., 2011). Thus, it is conceivable that neuropeptides regulate fear expression indirectly by affecting the activity of the CEl neurons that inhibit CEm neurons, and also by directly acting on CEm neurons (Huber et al., 2005). While the exact mechanisms remain to be determined, inhibitory circuits likely integrate neuropeptide signals in response to updated cues and in turn, modulate fear behavior.

## CONCLUSION

In the past several years there have been tremendous advances in the understanding of the functional roles that the inhibitory circuits in the amygdala play during acquisition and expression of conditioned fear and fear extinction. However, the multiple cell types that comprise these circuits and the complex connections of various pathways did make it difficult to determine the precise cellular and molecular mechanisms and to define the functional consequences of the circuitry for behavioral phenotypes. Therefore, cell type-specific stimulation of different types of interneurons, through usage of Cre driver mouse lines as well as optogenetic tools, will facilitate elucidation of the mechanisms underlying fear memories. Nevertheless, most of the current understanding of inhibitory circuits is based on a working hypothesis that the functions of these circuits include controlling cellular excitability and modulating synaptic plasticity. However, emerging evidence suggests that inhibitory components play critical roles in acquisition and behavioral expression of fear-related information through active participation in both intra- and extra-amygdala fear pathways that are important for storage of new memories and extinction of existing memories. Future work will be necessary to further delineate the roles of inhibitory circuits and provide a clearer picture for how fear memory works.

## Conflict of Interest Statement

The authors declare that the research was conducted in the absence of any commercial or financial relationships that could be construed as a potential conflict of interest.

## References

[B1] Allen Institute for Brain Science. (2012) *Allen Brain Atlas [Internet]*. Available at: (accessed December 27, 2012)

[B2] Amano T. Duvarci S. Popa D. Pare D. (2011) The fear circuit revisited: contributions of the basal amygdala nuclei to conditioned fear. *J. Neurosci.* 31 15481–15489 10.1523/JNEUROSCI.3410-11.2011 22031894 PMC3221940

[B3] Amano T. Unal C. T. Pare D. (2010) Synaptic correlates of fear extinction in the amygdala. *Nat. Neurosci.* 13 489–494 10.1038/nn.2499 20208529 PMC2847017

[B4] Amico J. A. Mantella R. C. Vollmer R. R. Li X. (2004) Anxiety and stress responses in female oxytocin deficient mice. *J. Neuroendocrinol.* 16 319–324 10.1111/j.0953-8194.2004.01161.x 15089969

[B5] Amorapanth P. LeDoux J. E. Nader K. (2000) Different lateral amygdala outputs mediate reactions and actions elicited by a fear-arousing stimulus. *Nat. Neurosci.* 3 74–79 10.1038/71145 10607398

[B6] An B. Hong I. Choi S. (2012) Long-term neural correlates of reversible fear learning in the lateral amygdala. *J. Neurosci.* 32 16845–16856 10.1523/JNEUROSCI.3017-12.2012 23175837 PMC6621751

[B7] Ascoli G. A. Alonso-Nanclares L. Anderson S. A. Barrionuevo G. Benavides-Piccione R. Burkhalter A. (2008) Petilla terminology: nomenclature of features of GABAergic interneurons of the cerebral cortex. *Nat. Rev. Neurosci.* 9 557–568 10.1038/nrn2402 18568015 PMC2868386

[B8] Aston-Jones G. Cohen J. D. (2005) An integrative theory of locus coeruleus-norepinephrine function: adaptive gain and optimal performance. *Annu. Rev. Neurosci.* 28 403–450 10.1146/annurev.neuro.28.061604.135709 16022602

[B9] Bergado-Acosta J. R. Sangha S. Narayanan R. T. Obata K. Pape H. C. Stork O. (2008) Critical role of the 65-kDa isoform of glutamic acid decarboxylase in consolidation and generalization of Pavlovian fear memory. *Learn. Mem.* 15 163–171 10.1101/lm.705408 18323571 PMC2275658

[B10] Bissiere S. Humeau Y. Luthi A. (2003) Dopamine gates LTP induction in lateral amygdala by suppressing feedforward inhibition. *Nat. Neurosci.* 6 587–592 10.1038/nn1058 12740581

[B11] Bouton M. E. (2004) Context and behavioral processes in extinction. *Learn. Mem.* 11 485–494 10.1101/lm.78804 15466298

[B12] Bouton M. E. Westbrook R. F. Corcoran K. A. Maren S. (2006) Contextual and temporal modulation of extinction: behavioral and biological mechanisms. *Biol. Psychiatry* 60 352–360 10.1016/j.biopsych.2005.12.015 16616731

[B13] Bowers M. E. Choi D. C. Ressler K. J. (2012) Neuropeptide regulation of fear and anxiety: implications of cholecystokinin, endogenous opioids, and neuropeptide Y. *Physiol. Behav.* 107 699–710 10.1016/j.physbeh.2012.03.004 22429904 PMC3532931

[B14] Brischoux F. Chakraborty S. Brierley D. I. Ungless M. A. (2009) Phasic excitation of dopamine neurons in ventral VTA by noxious stimuli. *Proc. Natl. Acad. Sci. U.S.A.* 106 4894–4899 10.1073/pnas.0811507106 19261850 PMC2660746

[B15] Burgos-Robles A. Vidal-Gonzalez I. Quirk G. J. (2009) Sustained conditioned responses in prelimbic prefrontal neurons are correlated with fear expression and extinction failure. *J. Neurosci.* 29 8474–8482 10.1523/JNEUROSCI.0378-09.2009 19571138 PMC2733220

[B16] Busti D. Geracitano R. Whittle N. Dalezios Y. Manko M. Kaufmann W. (2011) Different fear states engage distinct networks within the intercalated cell clusters of the amygdala. *J. Neurosci.* 31 5131–5144 10.1523/JNEUROSCI.6100-10.2011 21451049 PMC6622967

[B17] Carlsen J. (1988) Immunocytochemical localization of glutamate decarboxylase in the rat basolateral amygdaloid nucleus, with special reference to GABAergic innervation of amygdalostriatal projection neurons. *J. Comp. Neurol.* 273 513–526 10.1002/cne.902730407 3062049

[B18] Carrasco G. A Van de Kar L. D. (2003) Neuroendocrine pharmacology of stress. *Eur. J. Pharmacol.* 463 235–272 10.1016/S0014-2999(03)01285-8 12600714

[B19] Cassell M. D. Freedman L. J. Shi C. (1999) The intrinsic organization of the central extended amygdala. *Ann. N. Y. Acad. Sci.* 877 217–241 10.1111/j.1749-6632.1999.tb09270.x 10415652

[B20] Ciocchi S. Herry C. Grenier F. Wolff S. B. Letzkus J. J. Vlachos I. (2010) Encoding of conditioned fear in central amygdala inhibitory circuits. *Nature* 468 277–282 10.1038/nature09559 21068837

[B21] Corcoran K. A. Quirk G. J. (2007) Activity in prelimbic cortex is necessary for the expression of learned, but not innate, fears. *J. Neurosci.* 27 840–844 10.1523/JNEUROSCI.5327-06.2007 17251424 PMC6672908

[B22] Duvarci S. Popa D. Pare D. (2011) Central amygdala activity during fear conditioning. *J. Neurosci.* 31 289–294 10.1523/JNEUROSCI.4985-10.2011 21209214 PMC3080118

[B23] Ehrlich I. Humeau Y. Grenier F. Ciocchi S. Herry C. Luthi A. (2009) Amygdala inhibitory circuits and the control of fear memory. *Neuron* 62 757–771 10.1016/j.neuron.2009.05.026 19555645

[B24] Gilpin N. W. (2012) Corticotropin-releasing factor (CRF) and neuropeptide Y (NPY): effects on inhibitory transmission in central amygdala, and anxiety- & alcohol-related behaviors. *Alcohol* 46 329–337 10.1016/j.alcohol.2011.11.009 22560367 PMC3613993

[B25] Gilpin N. W. Roberto M. (2012) Neuropeptide modulation of central amygdala neuroplasticity is a key mediator of alcohol dependence. *Neurosci. Biobehav. Rev.* 36 873–888 10.1016/j.neubiorev.2011.11.002 22101113 PMC3325612

[B26] Goosens K. A. Maren S. (2001) Contextual and auditory fear conditioning are mediated by the lateral, basal, and central amygdaloid nuclei in rats. *Learn. Mem.* 8 148–155 10.1101/lm.37601 11390634 PMC311374

[B27] Goosens K. A. Maren S. (2003) Pretraining NMDA receptor blockade in the basolateral complex, but not the central nucleus, of the amygdala prevents savings of conditional fear. *Behav. Neurosci.* 117 738–750 10.1037/0735-7044.117.4.738 12931959

[B28] Greba Q. Gifkins A. Kokkinidis L. (2001) Inhibition of amygdaloid dopamine D2 receptors impairs emotional learning measured with fear-potentiated startle. *Brain Res.* 899 218–226 10.1016/S0006-8993(01)02243-0 11311883

[B29] Greba Q. Kokkinidis L. (2000) Peripheral and intraamygdalar administration of the dopamine D1 receptor antagonist SCH 23390 blocks fear-potentiated startle but not shock reactivity or the shock sensitization of acoustic startle. *Behav. Neurosci.* 114 262–272 10.1037/0735-7044.114.2.262 10832788

[B30] Griebel G. Stemmelin J. Gal C. S. Soubrie P. (2005) Non-peptide vasopressin V1b receptor antagonists as potential drugs for the treatment of stress-related disorders. *Curr. Pharm. Des.* 11 1549–1559 10.2174/1381612053764797 15892661

[B31] Guarraci F. A. Frohardt R. J. Falls W. A. Kapp B. S. (2000) The effects of intra-amygdaloid infusions of a D2 dopamine receptor antagonist on Pavlovian fear conditioning. *Behav. Neurosci.* 114 647–651 10.1037/0735-7044.114.3.647 10883814

[B32] Guarraci F. A. Frohardt R. J. Kapp B. S. (1999) Amygdaloid D1 dopamine receptor involvement in Pavlovian fear conditioning. *Brain Res.* 827 28–40 10.1016/S0006-8993(99)01291-3 10320690

[B33] Gulpinar M. A. Yegen B. C. (2004) The physiology of learning and memory: role of peptides and stress. *Curr. Protein Pept. Sci.* 5 457–473 10.2174/1389203043379341 15581416

[B34] Gutman A. R. Yang Y. Ressler K. J. Davis M. (2008) The role of neuropeptide Y in the expression and extinction of fear-potentiated startle. *J. Neurosci.* 28 12682–12690 10.1523/JNEUROSCI.2305-08.2008 19036961 PMC2621075

[B35] Hashimoto S. Inoue T. Koyama T. (1996) Serotonin reuptake inhibitors reduce conditioned fear stress-induced freezing behavior in rats. *Psychopharmacology (Berl.)* 123 182–186 10.1007/BF02246175 8741941

[B36] Haubensak W. Kunwar P. S. Cai H. Ciocchi S. Wall N. R. Ponnusamy R. (2010) Genetic dissection of an amygdala microcircuit that gates conditioned fear. *Nature* 468 270–276 10.1038/nature09553 21068836 PMC3597095

[B37] Heilig M. McLeod S. Brot M. Heinrichs S. C. Menzaghi F. Koob G. F. (1993) Anxiolytic-like action of neuropeptide Y: mediation by Y1 receptors in amygdala, and dissociation from food intake effects. *Neuropsychopharmacology* 8 357–363 10.1038/npp.1993.35 8099792

[B38] Herry C. Ciocchi S. Senn V. Demmou L. Muller C. Luthi A. (2008) Switching on and off fear by distinct neuronal circuits. *Nature* 454 600–606 10.1038/nature07166 18615015

[B39] Herry C. Ferraguti F. Singewald N. Letzkus J. J. Ehrlich I. Luthi A. (2010) Neuronal circuits of fear extinction. *Eur. J. Neurosci.* 31 599–612 10.1111/j.1460-9568.2010.07101.x 20384807

[B40] Hobin J. A. Goosens K. A. Maren S. (2003) Context-dependent neuronal activity in the lateral amygdala represents fear memories after extinction. *J. Neurosci.* 23 8410–8416 12968003 10.1523/JNEUROSCI.23-23-08410.2003 PMC2291151

[B41] Holmes A. Heilig M. Rupniak N. M. Steckler T. Griebel G. (2003) Neuropeptide systems as novel therapeutic targets for depression and anxiety disorders. *Trends Pharmacol. Sci.* 24 580–588 10.1016/j.tips.2003.09.011 14607081

[B42] Huber D. Veinante P. Stoop R. (2005) Vasopressin and oxytocin excite distinct neuronal populations in the central amygdala. *Science* 308 245–248 10.1126/science.1105636 15821089

[B43] Inglis F. M. Moghaddam B. (1999) Dopaminergic innervation of the amygdala is highly responsive to stress. *J. Neurochem.* 72 1088–1094 10.1046/j.1471-4159.1999.0721088.x 10037480

[B44] Jungling K. Seidenbecher T. Sosulina L. Lesting J. Sangha S. Clark S. D. (2008) Neuropeptide S-mediated control of fear expression and extinction: role of intercalated GABAergic neurons in the amygdala. *Neuron* 59 298–310 10.1016/j.neuron.2008.07.002 18667157 PMC2610688

[B45] Kim J. Lee S. Park K. Hong I. Song B. Son G. (2007) Amygdala depotentiation and fear extinction. *Proc. Natl. Acad. Sci. U.S.A.* 104 20955–20960 10.1073/pnas.0710548105 18165656 PMC2409248

[B46] Knobloch H. S. Charlet A. Hoffmann L. C. Eliava M. Khrulev S. Cetin A. H. (2012) Evoked axonal oxytocin release in the central amygdala attenuates fear response. *Neuron* 73 553–566 10.1016/j.neuron.2011.11.030 22325206

[B47] Kroner S. Rosenkranz J. A. Grace A. A. Barrionuevo G. (2005) Dopamine modulates excitability of basolateral amygdala neurons in vitro. *J. Neurophysiol.* 93 1598–1610 10.1152/jn.00843.2004 15537813

[B48] LaLumiere R. T. Buen T. V. McGaugh J. L. (2003) Post-training intra-basolateral amygdala infusions of norepinephrine enhance consolidation of memory for contextual fear conditioning. *J. Neurosci.* 23 6754–6758 12890768 10.1523/JNEUROSCI.23-17-06754.2003 PMC6740722

[B49] Lamont E. W. Kokkinidis L. (1998) Infusion of the dopamine D1 receptor antagonist SCH 23390 into the amygdala blocks fear expression in a potentiated startle paradigm. *Brain Res.* 795 128–136 10.1016/S0006-8993(98)00281-9 9622611

[B50] Lang E. J. Pare D. (1997) Similar inhibitory processes dominate the responses of cat lateral amygdaloid projection neurons to their various afferents. *J. Neurophysiol.* 77 341–352 9120575 10.1152/jn.1997.77.1.341

[B51] Lang E. J. Pare D. (1998) Synaptic responsiveness of interneurons of the cat lateral amygdaloid nucleus. *Neuroscience* 83 877–889 10.1016/S0306-4522(97)00420-X 9483571

[B52] Laurent V. Westbrook R. F. (2009) Inactivation of the infralimbic but not the prelimbic cortex impairs consolidation and retrieval of fear extinction. *Learn. Mem.* 16 520–529 10.1101/lm.1474609 19706835

[B53] Lazzaro S. C. Hou M. Cunha C. LeDoux J. E. Cain C. K. (2010) Antagonism of lateral amygdala alpha1-adrenergic receptors facilitates fear conditioning and long-term potentiation. *Learn. Mem.* 17 489–493 10.1101/lm.1918210 20870745 PMC2948893

[B54] LeDoux J. (2003) The emotional brain, fear, and the amygdala. *Cell. Mol. Neurobiol.* 23 727–738 10.1023/A:1025048802629 14514027 PMC11530156

[B55] LeDoux J. E. (2000) Emotion circuits in the brain. *Annu. Rev. Neurosci.* 23 155–184 10.1146/annurev.neuro.23.1.155 10845062

[B56] Ledoux J. E. Ruggiero D. A. Forest R. Stornetta R. Reis D. J. (1987) Topographic organization of convergent projections to the thalamus from the inferior colliculus and spinal cord in the rat. *J. Comp. Neurol.* 264 123–146 10.1002/cne.902640110 2445791

[B57] Li X. Inoue T. Abekawa T. Weng S. Nakagawa S. Izumi T. (2006) 5-HT1A receptor agonist affects fear conditioning through stimulations of the postsynaptic 5-HT1A receptors in the hippocampus and amygdala. *Eur. J. Pharmacol.* 532 74–80 10.1016/j.ejphar.2005.12.008 16460727

[B58] Li X. F. Armony J. L. LeDoux J. E. (1996a) GABAA and GABAB receptors differentially regulate synaptic transmission in the auditory thalamo-amygdala pathway: an in vivo microiontophoretic study and a model. *Synapse* 24 115–124 10.1002/(SICI)1098-2396(199610)24:2 8890453

[B59] Li X. F. Stutzmann G. E. LeDoux J. E. (1996b) Convergent but temporally separated inputs to lateral amygdala neurons from the auditory thalamus and auditory cortex use different postsynaptic receptors: in vivo intracellular and extracellular recordings in fear conditioning pathways. *Learn. Mem.* 3 229–242 10.1101/lm.3.2-3.229 10456093

[B60] Likhtik E. Pelletier J. G. Paz R. Pare D. (2005) Prefrontal control of the amygdala. *J. Neurosci.* 25 7429–7437 10.1523/JNEUROSCI.2314-05.2005 16093394 PMC6725290

[B61] Likhtik E. Popa D. Apergis-Schoute J. Fidacaro G. A. Pare D. (2008) Amygdala intercalated neurons are required for expression of fear extinction. *Nature* 454 642–645 10.1038/nature07167 18615014 PMC2528060

[B62] Lu Y. F. Moriwaki A. Tomizawa K. Onuma H. Cai X. H. Matsui H. (1997) Effects of vasopressin and involvement of receptor subtypes in the rat central amygdaloid nucleus in vitro. *Brain Res.* 768 266–272 10.1016/S0006-8993(97)00655-0 9369324

[B63] Maren S. Quirk G. J. (2004) Neuronal signalling of fear memory. *Nat. Rev. Neurosci.* 5 844–852 10.1038/nrn1535 15496862

[B64] Markram H. Toledo-Rodriguez M. Wang Y. Gupta A. Silberberg G. Wu C. (2004) Interneurons of the neocortical inhibitory system. *Nat. Rev. Neurosci.* 5 793–807 10.1038/nrn1519 15378039

[B65] Marowsky A. Yanagawa Y. Obata K. Vogt K. E. (2005) A specialized subclass of interneurons mediates dopaminergic facilitation of amygdala function. *Neuron* 48 1025–1037 10.1016/j.neuron.2005.10.029 16364905

[B66] Martina M. Royer S. Pare D. (1999) Physiological properties of central medial and central lateral amygdala neurons. *J. Neurophysiol.* 82 1843–1854 10515973 10.1152/jn.1999.82.4.1843

[B67] McCarthy M. M. McDonald C. H. Brooks P. J. Goldman D. (1996) An anxiolytic action of oxytocin is enhanced by estrogen in the mouse. *Physiol. Behav.* 60 1209–1215 10.1016/S0031-9384(96)00212-0 8916173

[B68] McDonald A. J. (1982) Cytoarchitecture of the central amygdaloid nucleus of the rat. *J. Comp. Neurol.* 208 401–418 10.1002/cne.902080409 7119168

[B69] McDonald A. J. (1998) Cortical pathways to the mammalian amygdala. *Prog. Neurobiol.* 55 257–332 10.1016/S0301-0082(98)00003-3 9643556

[B70] Milad M. R. Quirk G. J. (2002) Neurons in medial prefrontal cortex signal memory for fear extinction. *Nature* 420 70–74 10.1038/nature01138 12422216

[B71] Montezinho L. P. Miller S. Plath N. Jensen N. H. Karlsson J. J. Witten L. (2010) The effects of acute treatment with escitalopram on the different stages of contextual fear conditioning are reversed by atomoxetine. *Psychopharmacology (Berl.)* 212 131–143 10.1007/s00213-010-1917-5 20676614

[B72] Morozov A. Sukato D. Ito W. (2011) Selective suppression of plasticity in amygdala inputs from temporal association cortex by the external capsule. *J. Neurosci.* 31 339–345 10.1523/JNEUROSCI.5537-10.2011 21209220 PMC3080111

[B73] Nader K. Majidishad P. Amorapanth P. LeDoux J. E. (2001) Damage to the lateral and central, but not other, amygdaloid nuclei prevents the acquisition of auditory fear conditioning. *Learn. Mem.* 8 156–163 10.1101/lm.38101 11390635 PMC311372

[B74] Pare D. Duvarci S. (2012) Amygdala microcircuits mediating fear expression and extinction. *Curr. Opin. Neurobiol.* 22 717–723 10.1016/j.conb.2012.02.014 22424846 PMC3380167

[B75] Pare D. Smith Y. (1993) The intercalated cell masses project to the central and medial nuclei of the amygdala in cats. *Neuroscience* 57 1077–1090 10.1016/0306-4522(93)90050-P 8309544

[B76] Pitkanen A. Savander V. LeDoux J. E. (1997) Organization of intra-amygdaloid circuitries in the rat: an emerging framework for understanding functions of the amygdala. *Trends Neurosci.* 20 517–523 10.1016/S0166-2236(97)01125-9 9364666

[B77] Primeaux S. D. Wilson S. P. Bray G. A. York D. A Wilson M. A. (2006) Overexpression of neuropeptide Y in the central nucleus of the amygdala decreases ethanol self-administration in “anxious” rats. *Alcohol. Clin. Exp. Res.* 30 791–801 10.1111/j.1530-0277.2006.00092.x 16634847

[B78] Quirarte G. L. Galvez R. Roozendaal B. McGaugh J. L. (1998) Norepinephrine release in the amygdala in response to footshock and opioid peptidergic drugs. *Brain Res.* 808 134–140 10.1016/S0006-8993(98)00795-1 9767150

[B79] Quirk G. J. Repa C. LeDoux J. E. (1995) Fear conditioning enhances short-latency auditory responses of lateral amygdala neurons: parallel recordings in the freely behaving rat. *Neuron* 15 1029–1039 10.1016/0896-6273(95)90092-6 7576647

[B80] Raggenbass M. (2001) Vasopressin- and oxytocin-induced activity in the central nervous system: electrophysiological studies using in-vitro systems. *Prog. Neurobiol.* 64 307–326 10.1016/S0301-0082(00)00064-2 11240311

[B81] Rassnick S. Heinrichs S. C. Britton K. T. Koob G. F. (1993) Microinjection of a corticotropin-releasing factor antagonist into the central nucleus of the amygdala reverses anxiogenic-like effects of ethanol withdrawal. *Brain Res.* 605 25–32 10.1016/0006-8993(93)91352-S 8467387

[B82] Repa J. C. Muller J. Apergis J. Desrochers T. M. Zhou Y. LeDoux J. E. (2001) Two different lateral amygdala cell populations contribute to the initiation and storage of memory. *Nat. Neurosci.* 4 724–731 10.1038/89512 11426229

[B83] Rescorla R. A. (2001) Retraining of extinguished Pavlovian stimuli. *J. Exp. Psychol. Anim. Behav. Process.* 27 115–124 10.1037/0097-7403.27.2.115 11296487

[B84] Rescorla R. A. (2004) Spontaneous recovery. *Learn. Mem.* 11 501–509 10.1101/lm.77504 15466300

[B85] Rescorla R. A. Heth C. D. (1975) Reinstatement of fear to an extinguished conditioned stimulus. *J. Exp. Psychol. Anim. Behav. Process.* 1 88–96 10.1037/0097-7403.1.1.88 1151290

[B86] Rogan M. T. Staubli U. V. LeDoux J. E. (1997) Fear conditioning induces associative long-term potentiation in the amygdala. *Nature* 390 604–607 10.1038/37601 9403688

[B87] Rosenkranz J. A. Grace A. A. (2002) Dopamine-mediated modulation of odour-evoked amygdala potentials during pavlovian conditioning. *Nature* 417 282–287 10.1038/417282a 12015602

[B88] Royer S. Martina M. Pare D. (1999) An inhibitory interface gates impulse traffic between the input and output stations of the amygdala. *J. Neurosci.* 19 10575–10583 10575053 10.1523/JNEUROSCI.19-23-10575.1999 PMC6782425

[B89] Royer S. Martina M. Pare D. (2000) Polarized synaptic interactions between intercalated neurons of the amygdala. *J. Neurophysiol.* 83 3509–3518 10848566 10.1152/jn.2000.83.6.3509

[B90] Sadikot A. F. Parent A. (1990) The monoaminergic innervation of the amygdala in the squirrel monkey: an immunohistochemical study. *Neuroscience* 36 431–447 10.1016/0306-4522(90)90439-B 1977101

[B91] Samson R. D. Pare D. (2005) Activity-dependent synaptic plasticity in the central nucleus of the amygdala. *J. Neurosci.* 25 1847–1855 10.1523/JNEUROSCI.3713-04.2005 15716421 PMC6725937

[B92] Schiess M. C. Callahan P. M. Zheng H. (1999) Characterization of the electrophysiological and morphological properties of rat central amygdala neurons in vitro. *J. Neurosci. Res.* 58 663–673 10.1002/(SICI)1097-4547(19991201)58:5 10561694

[B93] Shaban H. Humeau Y. Herry C. Cassasus G. Shigemoto R. Ciocchi S. (2006) Generalization of amygdala LTP and conditioned fear in the absence of presynaptic inhibition. *Nat. Neurosci.* 9 1028–1035 10.1038/nn1732 16819521

[B94] Sierra-Mercado D. Padilla-Coreano N. Quirk G. J. (2011) Dissociable roles of prelimbic and infralimbic cortices, ventral hippocampus, and basolateral amygdala in the expression and extinction of conditioned fear. *Neuropsychopharmacology* 36 529–538 10.1038/npp.2010.184 20962768 PMC3005957

[B95] Smith Y. Pare D. (1994) Intra-amygdaloid projections of the lateral nucleus in the cat: PHA-L anterograde labeling combined with postembedding GABA and glutamate immunocytochemistry. *J. Comp. Neurol.* 342 232–248 10.1002/cne.903420207 7911130

[B96] Sotres-Bayon F. Sierra-Mercado D. Pardilla-Delgado E. Quirk G. J. (2012) Gating of fear in prelimbic cortex by hippocampal and amygdala inputs. *Neuron* 76 804–812 10.1016/j.neuron.2012.09.028 23177964 PMC3508462

[B97] Stutzmann G. E. LeDoux J. E. (1999) GABAergic antagonists block the inhibitory effects of serotonin in the lateral amygdala: a mechanism for modulation of sensory inputs related to fear conditioning. *J. Neurosci. * 19 RC8. 10.1523/JNEUROSCI.19-11-j0005.1999 PMC6782604 10341269

[B98] Stutzmann G. E. McEwen B. S. LeDoux J. E. (1998) Serotonin modulation of sensory inputs to the lateral amygdala: dependency on corticosterone. *J. Neurosci.* 18 9529–9538 9801389 10.1523/JNEUROSCI.18-22-09529.1998 PMC6792882

[B99] Szinyei C. Heinbockel T. Montagne J. Pape H. C. (2000) Putative cortical and thalamic inputs elicit convergent excitation in a population of GABAergic interneurons of the lateral amygdala. *J. Neurosci.* 20 8909–8915 11102501 10.1523/JNEUROSCI.20-23-08909.2000 PMC6773053

[B100] Tanaka M. Yoshida M. Emoto H. Ishii H. (2000) Noradrenaline systems in the hypothalamus, amygdala and locus coeruleus are involved in the provocation of anxiety: basic studies. *Eur. J. Pharmacol.* 405 397–406 10.1016/S0014-2999(00)00569-0 11033344

[B101] Tasan R. O. Nguyen N. K. Weger S. Sartori S. B. Singewald N. Heilbronn R. (2010) The central and basolateral amygdala are critical sites of neuropeptide Y/Y2 receptor-mediated regulation of anxiety and depression. *J. Neurosci.* 30 6282–6290 10.1523/JNEUROSCI.0430-10.2010 20445054 PMC3073168

[B102] Tully K. Li Y. Tsvetkov E. Bolshakov V. Y. (2007) Norepinephrine enables the induction of associative long-term potentiation at thalamo-amygdala synapses. *Proc. Natl. Acad. Sci. U.S.A.* 104 14146–14150 10.1073/pnas.0704621104 17709755 PMC1955781

[B103] Turner B. H. Herkenham M. (1991) Thalamoamygdaloid projections in the rat: a test of the amygdala’s role in sensory processing. *J. Comp. Neurol.* 313 295–325 10.1002/cne.903130208 1765584

[B104] Tye K. M. Prakash R. Kim S. Y. Fenno L. E. Grosenick L. Zarabi H. (2011) Amygdala circuitry mediating reversible and bidirectional control of anxiety. *Nature* 471 358–362 10.1038/nature09820 21389985 PMC3154022

[B105] Vertes R. P. (2004) Differential projections of the infralimbic and prelimbic cortex in the rat. *Synapse* 51 32–58 10.1002/syn.10279 14579424

[B106] Vidal-Gonzalez I. Vidal-Gonzalez B. Rauch S. L. Quirk G. J. (2006) Microstimulation reveals opposing influences of prelimbic and infralimbic cortex on the expression of conditioned fear. *Learn. Mem.* 13 728–733 10.1101/lm.306106 17142302 PMC1783626

[B107] Viviani D. Charlet A. van den Burg E. Robinet C. Hurni N. Abatis M. (2011) Oxytocin selectively gates fear responses through distinct outputs from the central amygdala. *Science* 333 104–107 10.1126/science.1201043 21719680

[B108] Wilensky A. E. Schafe G. E. Kristensen M. P. LeDoux J. E. (2006) Rethinking the fear circuit: the central nucleus of the amygdala is required for the acquisition, consolidation, and expression of Pavlovian fear conditioning. *J. Neurosci.* 26 12387–12396 10.1523/JNEUROSCI.4316-06.2006 17135400 PMC6674909

[B109] Wiltgen B. J. Godsil B. P. Peng Z. Saab F. June H. L. Linn M. L. (2009) The alpha1 subunit of the GABA(A) receptor modulates fear learning and plasticity in the lateral amygdala. *Front. Behav. Neurosci. * 3: 37 10.3389/neuro.08.037.2009 PMC2769557 19876409

[B110] Woodruff A. R. Sah P. (2007) Inhibition and synchronization of basal amygdala principal neuron spiking by parvalbumin-positive interneurons. *J. Neurophysiol.* 98 2956–2961 10.1152/jn.00739.2007 17715201

[B111] Yokoyama M. Suzuki E. Sato T. Maruta S. Watanabe S. Miyaoka H. (2005) Amygdalic levels of dopamine and serotonin rise upon exposure to conditioned fear stress without elevation of glutamate. *Neurosci. Lett.* 379 37–41 10.1016/j.neulet.2004.12.047 15814195

[B112] Zimmerman J. M. Rabinak C. A. McLachlan I. G. Maren S. (2007) The central nucleus of the amygdala is essential for acquiring and expressing conditional fear after overtraining. *Learn. Mem.* 14 634–644 10.1101/lm.607207 17848503 PMC1994080

[B113] Zweifel L. S. Fadok J. P. Argilli E. Garelick M. G. Jones G. L. Dickerson T. M. (2011) Activation of dopamine neurons is critical for aversive conditioning and prevention of generalized anxiety. *Nat. Neurosci.* 14 620–626 10.1038/nn.2808 21499253 PMC3083461

